# Diabetes Remission after Nonsurgical Intensive Lifestyle Intervention in Obese Patients with Type 2 Diabetes

**DOI:** 10.1155/2015/468704

**Published:** 2015-05-31

**Authors:** Adham Mottalib, Mahmoud Sakr, Mohamed Shehabeldin, Osama Hamdy

**Affiliations:** Joslin Diabetes Center, One Joslin Place, Boston, MA 02215, USA

## Abstract

Partial or complete remission from type 2 diabetes was recently observed after bariatric surgeries. Limited data is available about the possibility of inducing diabetes remission through intensive weight reduction. We retrospectively evaluated diabetes remissions after one year of the Weight Achievement and Intensive Treatment (Why WAIT) program, a 12-week intensive program for diabetes weight management in real-world clinical practice. Among 120 obese patients with type 2 diabetes who completed the program, 88 patients returned for follow-up at one year. Nineteen patients (21.6%) had major improvement in their glycemic control, defined as achieving an A1C <6.5% after one year. Four patients (4.5%) achieved either partial or complete diabetes remission defined as A1C <6.5% and <5.7%, respectively, on no antihyperglycemic medications for one year; 2 achieved partial remission (2.3%) and 2 achieved complete remission (2.3%). At the time of intervention, patients who achieved diabetes remission had shorter diabetes duration (<5 years) and lower A1C (<8%) and were treated with fewer than 2 oral medications. They achieved a weight reduction of >7% after 12 weeks. These results indicate that a subset of obese patients with type 2 diabetes is appropriate for intensive lifestyle intervention with the aim of inducing diabetes remission.

## 1. Introduction

Type 2 diabetes has been considered as an incurable chronic disease. Traditionally, the focus of clinical management has been directed toward controlling hyperglycemia and its consequent complications [1]. However, recent data from several studies, primarily surgical, showed that remission from diabetes may occur after significant weight loss [2, 3].

Since weight gain plays a vital role in the pathophysiology of type 2 diabetes, especially among genetically susceptible individuals [4], there is an urgent need to reexamine the actual value of weight loss as a major component of type 2 diabetes management. One meta-analysis showed that 60–90% of type 2 diabetes appears to be related to obesity or weight gain and that weight loss of 10% of initial body weight dramatically improves glycemic control and reduces other comorbid risk factors [5]. We also demonstrated that modest weight reduction of about 7% over 6-month period through caloric reduction and increased physical activity improves insulin sensitivity, endothelial function, and several markers of inflammation and coagulation in obese patients with and without diabetes [6, 7].

Another meta-analysis of long-term effects of weight loss on diabetes prevention among obese people showed that intentional weight loss reduces risk of developing diabetes by 25%, while larger weight loss achieved through surgical interventions showed a dramatic risk reduction of about 63% [8].

Bariatric surgery has been reported to improve type 2 diabetes and was shown to be superior to medical intervention [2, 9].

The aim of this study is to evaluate diabetes remission after one year of intensive lifestyle intervention in real-world clinical practice. It also aimed at characterizing those patients who are likely to achieve remission after weight loss.

## 2. Methods

### 2.1. Study Design

We retrospectively evaluated obese patients (BMI ≥ 30 kg/m^2^) with type 2 diabetes enrolled in the Weight Achievement and Intensive Treatment (Why-WAIT) program between September 2005 and June 2008. Why WAIT program is a 12-week multidisciplinary intensive weight management program customized for obese patients with diabetes in real-world clinical practice at the Joslin Diabetes Center in Boston [10]. Only patients with type 2 diabetes who completed one year of follow-up were included in this analysis. The data collected at baseline and after one year include body weight, absolute and percentage weight reduction, %A1C, blood pressure, lipid profile, serum creatinine, and the number of antihyperglycemic medications. Complete diabetes remission was defined by the American Diabetes Association 2009 consensus statement as a return to “normal” measures of glucose metabolism (A1C < 5.7, fasting glucose < 100 mg/dL) for at least one year in absence of active pharmacologic therapy or ongoing procedures. Partial remission was defined as hyperglycemia below the diagnostic level for diabetes (A1C < 6.5%, fasting glucose 100–125 mg/dL) for at least one year in absence of active pharmacologic therapy or ongoing procedures [11]. In this study, we defined major diabetes improvement as achieving A1C of <6.5% on single antihyperglycemic medication.

### 2.2. Intervention Method

A full description of the Why WAIT program was previously published [12]. The key components of the program include (1) intensive and interactive medication adjustments; (2) structured modified dietary intervention; (3) graded, balanced, and individualized exercise intervention; (4) cognitive behavioral support; and (5) adult group education. Participants were enrolled in groups of 12–15 individuals each. Each 2-hour weekly intervention session includes demonstrations of exercise at Joslin gymnasium, review of food and exercise logs, review of blood glucose log, adjustment of diabetes medications plus two 30-minute behavioral and didactics sessions. After completion of the program, participants were encouraged to come for monthly follow-up visits.

## 3. Results

**Figure 1:**
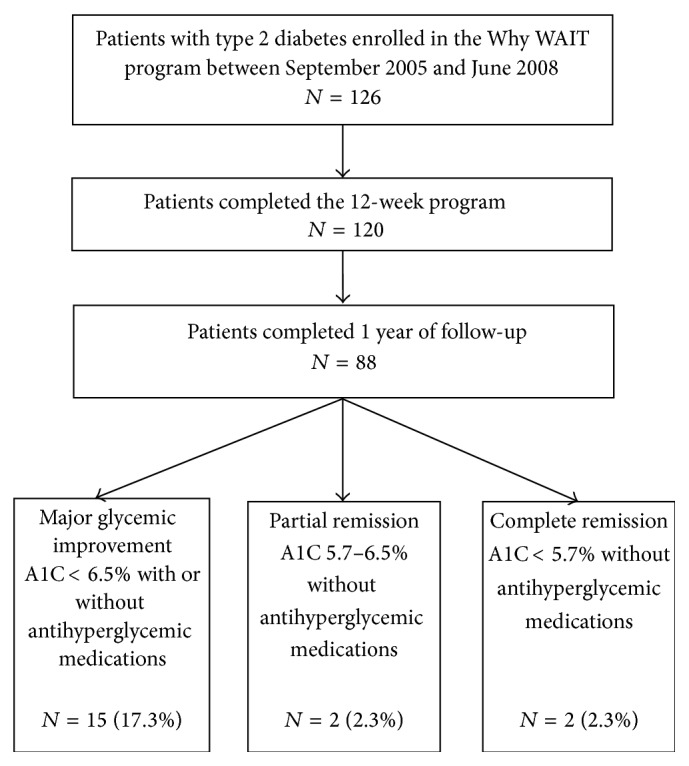
Flow of study participants.

**Figure 2:**
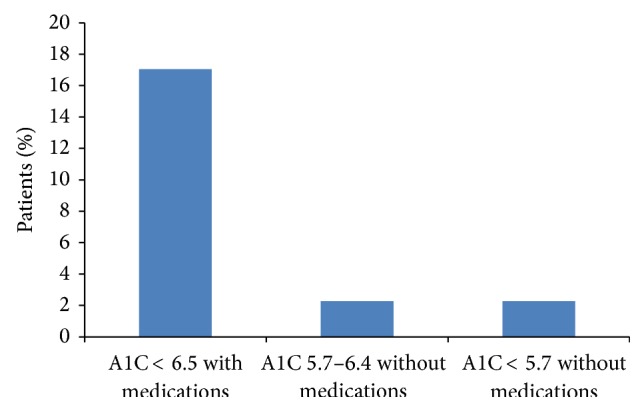
Percentage of participants who achieved major glycemic improvement, partial or complete diabetes remission at one year.

A total of 126 obese patients with type 2 diabetes were enrolled in the Why WAIT program during the study period. Six of them dropped out during the program for personal reasons. Out of the remaining 120 patients, 88 patients completed one year of follow-up (Figure 1). Nineteen participants (21.6%) had major improvement of glycemic control, 4 participants (4.6%) achieved either partial or complete diabetes remission, 2 achieved partial remission (2.3%), and 2 achieved complete remission (2.3%) (Figure 2).

Participants who achieved major glycemic improvement at one year had a mean age of 53.3 (±8.5) years, a mean body weight of 237.7 (±29.5) pounds, a BMI of 38.2 (±4) kg/m^2^, and a mean duration of diabetes of 5.1 (±5.6) years at the start of the Why WAIT program. They were treated with a mean of 1.5 antihyperglycemic medications and they lost on average 26.4 lbs (±8.5) (11.1%) by the end of the program and maintained an average weight loss of 26.6 lbs (±20.7) (11.2%) at one year. The mean change in A1C, body weight, BMI, blood pressure, lipid profile, and renal function were shown in Table 1.

Those who achieved partial or complete remission had a mean baseline body weight of 230.9 (±23.2) pounds, a BMI of 35.6 (±1.7) kg/m^2^, and a mean duration of diabetes of 1.5 (±0.9) years at the start of the program. They were treated with a mean of 1.25 antihyperglycemic medications and lost on average 25.2 lbs (±7.3) (10.9%) by the end of the program and maintained a mean weight loss of 15.9 lbs (±10.7) (6.9%) at one year.

## 4. Discussion

In this study we demonstrated that major improvement in glycemic control can be achieved and maintained for one year after nonsurgical intensive lifestyle intervention in real-world clinical practice. Partial or complete diabetes remission at one year was achieved in small percentage of patients. Remission was more likely to occur in patients with shorter duration of diabetes, on few oral antihyperglycemic medications and with A1C < 8% at start of intervention. These results may indicate that a subset of obese patients with type 2 diabetes on antihyperglycemic medications is appropriate for intensive lifestyle intervention that aims at inducing diabetes remission and stopping the use of antihyperglycemic medications.

Look AHEAD (Action for Health for Diabetes) study also showed that complete remission of type 2 diabetes after intensive lifestyle intervention is possible but rare [13, 14]. For instance, a mean reduction of body weight of about 9% after one year in the intensive lifestyle intervention arm was associated with a complete remission of only 1.3% of participants [15].

The concept of diabetes remission in established patients who are already treated with antihyperglycemic medications is intriguing as it contradicts the traditional concept that type 2 diabetes is a chronic progressive disease characterized by significant loss of beta-cell function (up to 80%) at the time of diagnosis [16, 17]. Bergman et al. originally challenged this concept in obese patients with type 2 diabetes although he confirmed its existence in lean patients. Using the minimal model calculation method to estimate beta-cell function and insulin sensitivity, he demonstrated that the prime pathogenesis of type 2 diabetes in obese patients is predominantly related to significant reduction in insulin action due to insulin resistance while the contribution of beta-cell dysfunction is minimal if any [18]. Several studies showed that beta-cell function is partially or fully restored after improving insulin sensitivity [19, 20]. We also previously demonstrated that that 7% weight reduction improves insulin sensitivity by around 57% as calculated through the same minimal model [7].

The other explanation for our findings is that patients with short diabetes duration may have significant beta-cell reserve more than what was originally thought. It is unlikely that diabetes remission occurs if 50% or more of beta-cell function is lost at the time of diagnosis. It is worth mentioning that the United Kingdom Prospective Diabetes Study (UKPDS), which created that concept, was not originally designed to evaluate beta-cell function [21]. The study used a rough technique (HOMA calculation) to retrospectively calculate beta-cell function and create a hypothetical graph that indicates a progressive decline of beta-cell function [17]. The impossibility of acquiring pancreatic biopsies from living individuals with type 2 diabetes limits our knowledge about the actual beta-cell volume and function in living humans. Although several indirect methods have been developed over years, they all have major limitations [22].

This study also demonstrated several indicators of possible diabetes remission in response to intensive lifestyle intervention. They include short duration of diabetes of <5 years, A1C < 8% at the time of intervention, and treatment with fewer than 2 oral medications with higher weight loss of >7% as predictor of improvement. Surgical weight loss studies also showed that patients with short diabetes duration are more likely to achieve diabetes remission after surgery [2].

The current widely accepted concept is that oral antihyperglycemic medications are needed after failure of lifestyle intervention in controlling hyperglycemia [23]. However, growing evidence, including this study, indicates that lifestyle intervention plays a prime role in diabetes management and the effort to implement it should be more than the general advice to lose weight and to increase physical activity. Early diabetes management through intensive lifestyle intervention carries lower risk of hypoglycemia and lower cost of diabetes management than early use of antihyperglycemic medications [24]. Although bariatric surgeries showed significant higher rates of diabetes remission, these surgeries continue to pose short- and long-term risk of high comorbidity [25]. Absence of enough long-term follow-up data for patients that underwent bariatric surgeries suggests that bariatric surgery probably should not be the first-line therapy as advocated by some bariatric surgeons [2, 26]. A recent study by Blue Cross and Blue Shield challenged the cost-effectiveness of bariatric surgery where health care cost 6 years after surgery was higher than the health care cost before surgery due to higher rate of outpatients visits and hospitalization [27].

This study has several limitations. It is a retrospective observational study. The study also included heterogeneous group of patients with variable diabetes duration and on variable number of antihyperglycemic medications including insulin. These limitations impacted significantly the rate of remission since it is unlikely that patients with long diabetes duration achieve diabetes remission after significant weight reduction. The Look AHEAD study, despite being a randomized controlled study, was not designed or powered to evaluate diabetes remission [13]. However the strength of this study over the Look AHEAD is that it was conducted in real-world clinical practice. This study indicates the possible benefit of a randomized controlled prospective study that is designed specifically to evaluate diabetes remission. It may be of value to target patients with similar characteristics to those who achieved diabetes remission in this study.

## Figures and Tables

**Table 1:** Changes in cardiovascular risk factors after 12 weeks of intensive lifestyle intervention and at one year in participants who achieved major glycemic improvement and partial or complete diabetes remission.

	Baseline	3 Months	12 months
Weight (lbs)	237.7 ± 29.5	211.3 ± 26.1^*∗∗*^	211 .1 ± 26.5^*∗∗*^
BMI (kg/m^2^)	38.2 ± 4	34.0 ± 3.8^*∗∗*^	40.0 ± 4.2^*∗∗*^
Systolic BP (mmHg)	128 ± 11	122 ± 15^*∗*^	124 ± 16
Diastolic BP (mmHg)	73 ± 8	74 ± 8	73 ± 9
Total cholesterol (mg/dL)	171 ± 31	147 ± 25^*∗∗*^	159 ± 33
LDL-cholesterol (mg/dL)	103 ± 23	88 ± 18^*∗∗*^	91 ± 24
HDL-cholesterol (mg/dL)	41 ± 10	40 ± 11	47 ± 13^*∗*^
Triglycerides (mg/dL)	144 ± 69	94 ± 41^*∗∗*^	123 ± 42

(*N* = 19).

^*∗*^ *p* value <0.05 from baseline, ^*∗∗*^ *p* value <0.001 from baseline.

## References

[1] Nathan D. M. (1993). Long-term complications of diabetes mellitus. *The New England Journal of Medicine*.

[2] Mingrone G., Panunzi S., de Gaetano A. (2012). Bariatric surgery versus conventional medical therapy for type 2 diabetes. *The New England Journal of Medicine*.

[3] Buchwald H., Estok R., Fahrbach K. (2009). Weight and type 2 diabetes after bariatric surgery: systematic review and meta-analysis. *The American Journal of Medicine*.

[4] Colditz G. A., Willett W. C., Rotnitzky A., Manson J. E. (1995). Weight gain as a risk factor for clinical diabetes mellitus in women. *Annals of Internal Medicine*.

[5] Anderson J. W., Kendall C. W. C., Jenkins D. J. A. (2003). Importance of weight management in Type 2 diabetes: review with meta-analysis of clinical studies. *Journal of the American College of Nutrition*.

[6] Hamdy O., Ledbury S., Mullooly C. (2003). Lifestyle modification improves endothelial function in obese subjects with the insulin resistance syndrome. *Diabetes Care*.

[7] Monzillo L. U., Hamdy O., Horton E. S. (2003). Effect of lifestyle modification on adipokine levels in obese subjects with insulin resistance. *Obesity Research*.

[8] Aucott L., Poobalan A., Smith W. C. S. (2004). Weight loss in obese diabetic and non-diabetic individuals and long-term diabetes outcomes—a systematic review. *Diabetes, Obesity and Metabolism*.

[9] Sjöström L., Lindroos A.-K., Peltonen M. (2004). Lifestyle, diabetes, and cardiovascular risk factors 10 years after bariatric surgery. *The New England Journal of Medicine*.

[10] Hamdy O. (2008). Diabetes weight management in clinical practice—the why wait model. *US Endocrinology*.

[11] Buse J. B., Caprio S., Cefalu W. T. (2009). How do we define cure of diabetes?. *Diabetes Care*.

[12] Hamdy O., Carver C. (2008). The why WAIT program: improving clinical outcomes through weight management in type 2 diabetes. *Current Diabetes Reports*.

[13] Gregg E. W., Chen H., Wagenknecht L. E. (2012). Association of an intensive lifestyle intervention with remission of type 2 diabetes. *Journal of the American Medical Association*.

[14] Wing R. R., Bahnson J. L., Bray G. A. (2010). Long-term effects of a lifestyle intervention on weight and cardiovascular risk factors in individuals with type 2 diabetes mellitus: four-year results of the look AHEAD trial. *Archives of Internal Medicine*.

[15] Gregg E. W., Kyithar M. P., Dinneen S. F. (2013). Acp journal club: an intensive lifestyle intervention increased remission from type 2 diabetes in overweight adults. *Annals of Internal Medicine*.

[16] Defronzo R. A. (2009). From the triumvirate to the ominous octet: a new paradigm for the treatment of type 2 diabetes mellitus. *Diabetes*.

[17] Turner R. C., Mann J. I., Holman R. R. (1988). UK prospective diabetes study. V. Characteristics of newly presenting type 2 diabetic patients: estimated insulin sensitivity and islet B-cell function. *Diabetic Medicine*.

[18] Bergman R. N., Phillips L. S., Cobelli C. (1981). Physiologic evaluation of factors controlling glucose tolerance in man. Measurement of insulin sensitivity and *β*-cell glucose sensitivity from the response to intravenous glucose. *The Journal of Clinical Investigation*.

[19] Kahn S. E., Prigeon R. L., McCulloch D. K. (1993). Quantification of the relationship between insulin sensitivity and *β*-cell function in human subjects: evidence for a hyperbolic function. *Diabetes*.

[20] Xiang A. H., Peters R. K., Kjos S. L. (2004). Pharmacological treatment of insulin resistance at two different stages in the evolution of type 2 diabetes: impact on glucose tolerance and *β*-cell function. *The Journal of Clinical Endocrinology & Metabolism*.

[21] Turner R. C., Mann J. I., Iceton G. (1983). UK prospective study of therapies of maturity-onset diabetes. I. Effect of diet, sulphonylurea, insulin or biguanide therapy on fasting plasma glucose and body weight over one year. *Diabetologia*.

[22] Cernea S., Dobreanu M. (2013). Diabetes and beta cell function: from mechanisms to evaluation and clinical implications. *Biochemia Medica*.

[23] Nathan D. M., Buse J. B., Davidson M. B. (2009). Medical management of hyperglycemia in type 2 diabetes: a consensus algorithm for the initiation and adjustment of therapy: a consensus statement of the american diabetes association and the european association for the study of diabetes. *Diabetes Care*.

[24] (1995). Effect of intensive diabetes management on macrovascular events and risk factors in the diabetes control and complications trial. *The American Journal of Cardiology*.

[25] Sjöström L. (2008). Bariatric surgery and reduction in morbidity and mortality: experiences from the SOS study. *International Journal of Obesity*.

[26] Puzziferri N., Roshek T. B., Mayo H. G., Gallagher R., Belle S. H., Livingston E. H. (2014). Long-term follow-up after bariatric surgery: a systematic review. *The Journal of the American Medical Association*.

[27] Bleich S. N., Chang H. Y., Lau B. (2012). Impact of bariatric surgery on health care utilization and costs among patients with diabetes. *Medical Care*.

